# UNTANGLING THE MODEL MINORITY STEREOTYPE: PROMOTING HEALTH EQUITY AMONG ASIAN AMERICAN OLDER ADULTS

**DOI:** 10.1093/geroni/igad104.0480

**Published:** 2023-12-21

**Authors:** Bei Wu, Yuri Jang

## Abstract

The “model minority” stereotype portrays Asian Americans (AAs) as a monolithic group with common traits, e.g., high levels of education and income, and better health status. Five studies in this symposium used multiple data sources and provided rich information to untangling this stereotype. The first study highlighted racial/ethnic disparities in help-seeking behaviors among Americans with subjective cognitive decline, revealing that AAPIs exhibit the lowest likelihood of seeking help for cognitive problems. The second study examined the degree to which socioeconomic disparity contributes to functional impairment among older AAs relative to other racial/ethnic groups, revealing that AAs fare worst in the burden of functional impairment due to education disparity. The third paper explored the cultural adaptation of the NYUCI for Chinese and Korean dementia caregivers, showing that cultural beliefs have a profound effect on caregivers’ views of dementia and caregiving, their use of social and family support, and their understanding of self-care and counseling services. The fourth study assessed factors linked to depressive symptoms among older Southeast AAs, revealing that Vietnamese Americans have significantly more depressive symptoms than Indonesian and Filipino Americans. Resilience and age are negatively related to depressive symptoms, while greater family conflict and inability to speak English fluently are positively associated with depressive symptoms. The fifth study used a large longitudinal survey on older Chinese Americans and demonstrated that higher acculturation levels are associated with a more rapid decline in depression and a slower decline in activities of daily living but are not related to long-term cognitive changes.

